# Case Reports: Bronchial Mucosal Vasculature Is Also Involved in the Acute Vascular Distress Syndrome of COVID-19

**DOI:** 10.3389/fmed.2021.710992

**Published:** 2021-11-30

**Authors:** Vincent Jounieaux, Damien Basille, Bénédicte Toublanc, Claire Andrejak, Daniel Oscar Rodenstein, Yazine Mahjoub

**Affiliations:** ^1^Pneumology Department, University Hospital Centre, Amiens, France; ^2^Pneumology Department, Cliniques Universitaires Saint-Luc, Université Catholique de Louvain, Brussels, Belgium; ^3^Cardiac, Thoracic-vascular and Respiratory Intensive Care Unit, Department of Anesthesia and Critical Care, University Hospital Centre, Amiens, France

**Keywords:** SARS-CoV-2, bronchovideoscopy, NBI (narrow band imaging), intrapulmonary shunt, AVDS

## Abstract

**Background:** The severe acute respiratory syndrome coronavirus 2 (SARS-CoV-2) which targets the pulmonary vasculature is supposed to induce an intrapulmonary right to left shunt with an increased pulmonary blood flow. We report here what may be, to the best of our knowledge, the first videoendoscopic descriptions of an hypervascularization of the bronchial mucosa in two patients hospitalized for coronavirus disease 2019 (COVID-19) pneumonia.

**Cases Presentation:** Two patients, 27- and 37-year-old, were addressed to our Pneumology department for suspicion of COVID-19 pneumonia. Their symptoms (fever, dry cough, and dyspnoea), associated to pulmonary ground glass opacities on thoracic CT, were highly suggestive of a COVID-19 disease despite repeated negative pharyngeal swabs RT-PCR. In both patients, bronchoscopy examination using white light was unremarkable but NBI bronchoscopy revealed a diffuse hypervascularization of the mucosa from the trachea to the sub-segmental bronchi, associated with dilated submucosal vessels. RT-PCR performed in bronchoalveolar lavage (BAL) confirmed the presence of Sars-CoV-2.

**Conclusions:** These two case reports highlight the crucial importance of the vascular component of the viral disease. We suggest that such bronchial hypervascularization with dilated vessels contributes, at least in part, to the intrapulmonary right to left shunt that characterizes the COVID-19 related Acute Vascular Distress Syndrome (AVDS). The presence of diffuse bronchial hypervascularization in the context of COVID-19 pandemic should prompt the search for Sars-CoV-2 in BAL samples.

The presence of diffuse endobronchial submucosal vessels dilation and proliferation, not described until now in coronavirus disease 2019 (COVID-19) disease, was demonstrated using narrow band imaging during diagnostic bronchoscopy in two patients with negative pharyngeal swabs RT-PCR. This unusual finding may prove of value in assessing patients without confirmatory laboratory data. These two case reports show how narrow band imaging (NBI) can help in the diagnosis of COVID-19 disease by showing diffuse endobronchial submucosal vessels dilatation and proliferation and demonstrating an unknown bronchoscopic vascular aspect of COVID-19-related Acute Vascular Distress Syndrome (AVDS).

## Case Report #1

**Figure 1:**
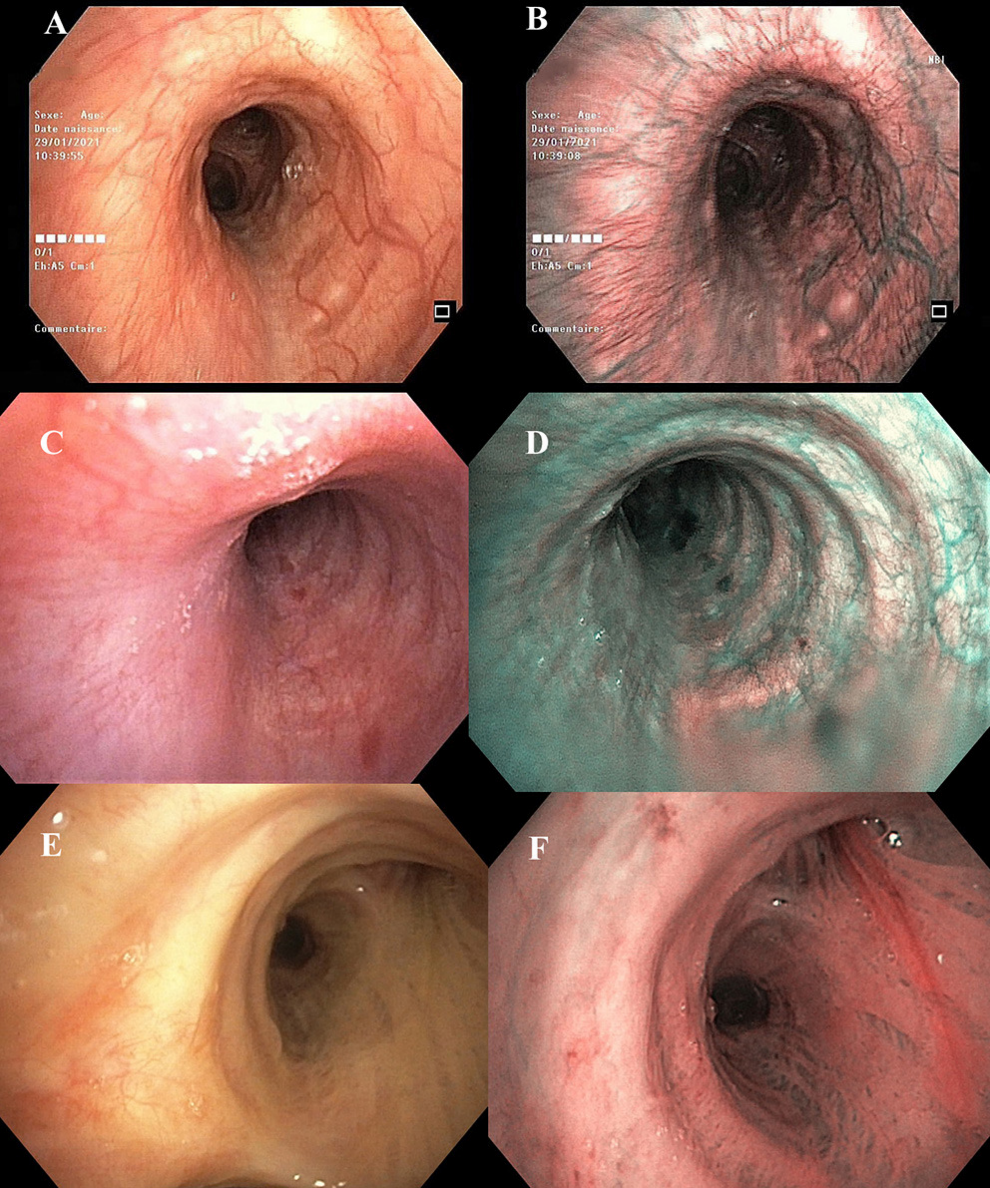
Case report patient #1–Left main bronchus **(A)** white-light, **(B)** Narrow band imaging (NBI) of the same region. Case report patient #2—Left main bronchus **(C)** white-light, **(D)** NBI of the same region. These images show in patients #1 and #2 a bronchial hypervascularization with dilated vessels. Note that in patient #1 the heavy dilated bronchial vessels developed on the axis of the bronchus, without any concomitant bronchial abnormalities. Patient #3—Right main bronchus **(E)** white-light, **(F)** NBI of the same region. These images show an example of the bronchial vascularization in a 77-year-old male patient hospitalized for a non-COVID-19 infection.

A 37-year-old man was hospitalized for suspicion of COVID-19 pneumonia on January 27, 2021. This non-smoker and athletic patient was only treated for hypertension (Ibesartan) and worked as a host in a nursing home where a COVID-19 cluster raged at this time. His symptoms began on January 17, with fever, dry cough, dyspnoea, headache, myalgia, diarrhea. In this context, the patient underwent two RT-PCR pharyngeal swabs (January 18, 2021 and January 21, 2021) that were negative for severe acute respiratory syndrome coronavirus 2 (SARS-CoV-2). The symptoms persisted despite a prescription of amoxicillin and he was addressed to our university hospital. The patient presented with fever (39°C), asthenia, headache, and dyspnea. On clinical examination the BMI was 26 kg/m^2^; blood pressure 135/80 mmHg; crackles were heard bilaterally, and the transcutaneous O2 saturation was 92%. Laboratory tests: WBC = 3,900 cells/mm^3^ (57% neutrophils, 32% lymphocytes), CRP: 14.7 mg/L, normal d-dimer value (0.38 μg/ml), arterial blood gas while breathing room air: pH, 7.45; PaO_2_, 59.9 mm Hg; and PaCO_2_, 36.4 mm Hg. Thoracic computed tomography (CT) showed mild evidence of pulmonary lesions related to COVID-19 (15% lung involvement by ground glass opacities). A third pharyngeal swab RT-PCR (January 27, 2021) was negative as well as the blood hemocultures. A diagnostic bronchoalveolar lavage (BAL) was proposed, and performed on January 28 (Olympus bronchovideoscope BF-H190, Tokyo, Japan), 12 days after the onset of symptoms. Bronchoscopy examination using white light (WL) was unremarkable without any abnormal aspect of the bronchial mucosa except a striking unusual presence of bronchial vessels. Narrow band imaging (NBI) bronchoscopy revealed a diffuse hypervascularization of the mucosa from the trachea to the sub-segmental bronchi, associated with dilated submucosal vessels which were developed on the axis of the bronchi (Figures 1A,B). Bronchoalveolar lavage (BAL) was performed in RB4 and its RT-PCR analysis confirmed the presence of SARS-CoV-2. The patient received cefotaxim and dexamethasone during 6 days. He became apyretic with an O_2_ saturation of 100% and returned home on February 2, 2021.

## Case Report #2

A 27-year-old man was hospitalized for suspicion of COVID-19 pneumonia on April 9, 2021. This non-smoker was only treated for hypertension for 10 years (Manidipine) and teleworked as a butcher's instructor. He was married and was the father of a 9-month child. His symptoms began on March 30 with asthenia, headache, and dizziness. The patient underwent a COVID-19 rapid test followed by a pharyngeal swab RT-PCR (April 1, 2021) which was negative for SARS-CoV-2. The symptoms worsened with diarrhea, dry cough, persistent fever despite a prescription of amoxicillin (April 7, 2021), and finally dyspnoea. He was then addressed to our university hospital. The patient presented with fever (38.4°C), diarrhea, and dyspnea at rest. On clinical examination the BMI was 29.8 kg/m^2^; blood pressure 140/78 mmHg; pulmonary auscultation was unremarkable and the transcutaneous O_2_ saturation was 93%. Laboratory tests: WBC = 7,800 cells/mm^3^ (85.5% neutrophils, 10.5% lymphocytes), CRP: 96.6 mg/L, d-dimers:.85 μg/ml, arterial blood gas while breathing room air: pH, 7.49; PaO_2_, 65 mm Hg; and PaCO_2_, 29.1 mm Hg. Thoracic computed tomography (CT) showed mild evidence of pulmonary lesions related to COVID-19 (10–25% lung involvement by ground glass opacities). A second COVID-19 rapid test was negative as well as the second pharyngeal swab RT-PCR (April 9, 2021). Blood hemocultures were negative. A diagnostic BAL was proposed and performed on April 10 (Olympus bronchovideoscope BF-H1100), 12 days after the onset of symptoms. Bronchoscopy examination using WL was unremarkable except the unusual presence of bronchial vessels. NBI bronchoscopy revealed a diffuse hypervascularization of the mucosa from the trachea to the sub-segmental bronchi, associated with dilated submucosal vessels (Figures 1C,D). BAL was performed in LB4 and its RT-PCR analysis confirmed the presence of SARS-CoV-2. The patient received cefotaxim for 5 days. He became apyretic with an O_2_ saturation of 98% and returned home on April 15, 2021.

## Discussion

These two cases reported present the first application on bronchovideoscopic NBI to patients with COVID-19, showing a diffuse hypervascularization of the mucosa from the trachea to the sub-segmental bronchi. NBI is generally used in malignant but also, and more, in premalignant airway lesions to detect focal bronchial hypervascularization. Observation of a diffuse hypervascularization with dilated submucosal vessels in patients with COVID-19 is a novel finding which supports the known neo-angiogenesis that leads to an intrapulmonary shunt during COVID-19 infection.

The circulation of the bronchial wall—composed of two different networks (mucosal and submucosal) connected by penetrating vessels—is essential to maintain the homeostasis by conditioning the inspired air (1). The bronchial vessels supply intrapulmonary airways as far as the terminal bronchioles to form anastomoses with the pulmonary vasculature. Two-thirds of the bronchial vein drainage return to the left atrium through the pulmonary veins whereas one- third returns to the right atrium through the azygous vein and superior vena cava (2). NBI is a recent endoscopic technique designed for the detection of pathologically altered submucosal and mucosal microvascular patterns. NBI uses two narrow-bands of light (400–430 and 525–550 nm, respectively). The blue narrow band (390–445 nm) is absorbed by surface mucosal layer capillaries whereas the green narrow band (530–550 nm) is absorbed by the hemoglobin in the deeper submucosal thick blood vessels (3). Their combination allows a detailed image of superficial bronchial vascularization. The combination of magnification video bronchoscopy and NBI has showed great potential in the detection of precancerous and cancerous lesions of the bronchial mucosa (4). NBI is able to detect the onset of angiogenesis during multi-step carcinogenesis of the lung and the meta-analysis by Iftikhar et al. has shown that NBI is better than autofluorescence imaging in the detection of premalignant airway lesions, showing a pooled sensitivity, specificity, and the diagnostic odds ratio of 80, 84, and 31.49%, respectively (3). The video bronchoscopic NBI aspect of the bronchial vascularization is clearly different in patients with COVID-19 and Figure 1 illustrates the differences when compared to the bronchial vascularization recorded in a 77-year-old male patient that underwent a video bronchoscopy for a non-COVID-19 infection. Based on the endoscopic classification of Shibuya et al. (5), the vascular lesions observed in our patients can be described with NBI as increased vessel growth, complex networks, and some dotted vessels. No tortuous vessels, small spiral or screw type vessels have been observed in our patients as described in angiogenic squamous dysplasia (ASD) or in carcinoma *in situ* (CIS). Because the video endoscopies with WL were normal in these two young non-smoker patients, the bronchial angiogenesis observed with NBI may be related to the ongoing COVID-19 infection. Indeed, it is now well-known that SARS-CoV-2 induces a neoangiogenesis and the histopathologic study of Ackermann et al. has clearly demonstrated in patients with COVID-19 the presence of pulmonary angiogenesis as early as day 4 of hospital admission and significantly increases with time (6). Chau et al. recently found that the median diameter of the bronchial artery at the origin was drastically increased in COVID-19 patients with pneumonia (7). This increase in bronchial circulation could promote the spread of inflammatory mediators throughout the lungs (2) and also increase the specific right to left shunt observed in patients with COVID-19. Indeed, during the first wave, we hypothesized that the cornerstone of COVID-19 disease was a vascular injury with an intrapulmonary shunt [as observed in the hepatopulmonary syndrome (8)] and proposed the acronym AVDS for Acute Vascular Distress Syndrome (9) to describe it. This initial hypothesis of AVDS was further supported by physiological approach and clinical observations (10–14) and reinforced by new imaging techniques (15).

Some limitations must be considered as these two observations, even provoking and illustrative, do not allow for any definitive conclusion. Thus, prospective studies are required to confirm the presence of a diffuse bronchial hypervascularization in patients with COVID-19 (through bronchovideoscopic NBI) and to precise its specificity when compared to non-COVID-19 patients. Indeed, objective tests are lacking that could have supported our hypothesis drawn from the videoendoscopic observations. Invasive investigations (mainly right-heart catheter or central venous catheter with measurements of arterial and venous oxygen contents allowing calculation of the right-to-left shunt) were not performed because our patients presented a non-severe COVID-19 infection and were not hospitalized in ICU. Moreover, dual energy lung CT scan was not available in our institution and data that could have shown pulmonary or bronchial dilated vessels is also lacking in our observations.

The COVID-19 related bronchial angiogenesis differs from those observed during carcinogenesis related angiogenesis. In these two case reports of a COVID-19 pneumonia, we observed a clear-cut bronchial hypervascularization with heavy dilated submucosal vessels developed along the axis of the main and lobar bronchi. These peculiarities, which may be specific of COVID-19 disease, could be interesting for definite diagnosis—in association with RT-PCR on BAL sample—in case of negative pharyngeal swabs RT-PCR.

## Data Availability Statement

The original contributions presented in the study are included in the article/supplementary material, further inquiries can be directed to the corresponding authors.

## Ethics Statement

Written informed consent was obtained from the individual(s) for the publication of any potentially identifiable images or data included in this article.

## Author Contributions

VJ performed the bronchovideoscopies, coordinated of the study and drafted the manuscript. VJ, DB, BT, and CA contributed to the management of these two patients and analyzed tests results. YM and DR contributed in interpretation of the data, in conducting the literature review, and writing the manuscript. All authors have read and approved the final manuscript.

## Conflict of Interest

The authors declare that the research was conducted in the absence of any commercial or financial relationships that could be construed as a potential conflict of interest.

## Publisher's Note

All claims expressed in this article are solely those of the authors and do not necessarily represent those of their affiliated organizations, or those of the publisher, the editors and the reviewers. Any product that may be evaluated in this article, or claim that may be made by its manufacturer, is not guaranteed or endorsed by the publisher.
